# Sp1, Instead of AhR, Regulates the Basal Transcription of Porcine *CYP1A1* at the Proximal Promoter

**DOI:** 10.3389/fphar.2018.00927

**Published:** 2018-08-17

**Authors:** Xuan Xie, Jun Jiang, Wenchu Ye, Ruohong Chen, Yiqun Deng, Jikai Wen

**Affiliations:** ^1^Guangdong Provincial Key Laboratory of Protein Function and Regulation in Agricultural Organisms, College of Life Sciences, South China Agricultural University, Guangzhou, China; ^2^Key Laboratory of Zoonosis of Ministry of Agriculture and Rural Affairs, South China Agricultural University, Guangzhou, China

**Keywords:** porcine, CYP1A1, Sp1, AhR, basal transactivation

## Abstract

Pigs are commonly used as an animal model to evaluate the toxic effects of exogenous compounds. Cytochrome P450 1A1 (CYP1A1) metabolizes numerous exogenous compounds and is abundantly expressed in the liver, kidneys, and intestines. The high amino acid similarity between human and porcine CYP1A1 indicates that they probably have the same metabolic characteristics. Therefore, understanding the regulatory mechanism of CYP1A1 expression in pigs is particularly important for predicting the toxicology and metabolic kinetics of exogenous chemicals. Currently, the transcriptional regulation of porcine *CYP1A1* has rarely been studied, especially regarding basal transcription. In this study, we first confirmed that the key regulatory elements of porcine *CYP1A1* basal transactivation are in the proximal promoter region using promoter truncation analysis via a dual luciferase assay in a porcine kidney cell line LLC-PK1. Two overlapping *cis*-elements, the xenobiotic response element (XRE) and GC box, in this proximal region potentially play key roles in the basal transactivation of porcine *CYP1A1*. Furthermore, using electrophoretic mobility shift assay and chromatin immunoprecipitation, the GC box binding protein Sp1 was confirmed to bind to the proximal promoter of porcine *CYP1A1*, instead of AhR, the XRE binding protein. In LLC-PK1 cells, by knocking down either Sp1 or AhR, the expression of porcine *CYP1A1* at the mRNA level and protein level was significantly downregulated, suggesting both proteins are important for porcine *CYP1A1* expression. However, promoter activity analysis in LLC-PK1 cells treated with an AhR agonist and antagonist confirmed that AhR does not participate in the basal regulation of porcine *CYP1A1* at the proximal promoter. In conclusion, our study revealed that the proximal promoter is the key regulatory region for porcine *CYP1A1* basal expression. Although AhR plays an important role in the transactivation of porcine *CYP1A1* expression, the key determinant transcription factor for its basal transactivation is Sp1 at the proximal promoter of porcine *CYP1A1*.

## Introduction

CYP1A1 is one of the most important CYP450 family members for metabolizing multiple exogenous and endogenous substrates such as drugs, carcinogens, steroids, and toxicants (Guengerich et al., 1991; Gray and Squires, 2013). The expression of CYP1A1 is correlated with carcinogen bioactivation (Guengerich, 1988) and the biotransformation of various chemical substrates by oxygenation, demethylation, or dealkylation. The pig is a valuable animal model as a substitute for humans in pharmacological and toxicological studies (Kojima and Degawa, 2016). Porcine CYP1A1 (pCYP1A1) is the orthologous protein to human CYP1A1, which plays a key role in the metabolism of various toxic and carcinogenic compounds (Ma and Lu, 2007). Therefore, clarifying the regulation of *CYP1A1* expression in pigs is instructive in understanding the metabolism of various drugs in humans.

CYP1A1 and 1A2 are highly induced by many chemicals at the mRNA and protein levels in different cells. However, the basal transcription mechanism remains to be further elucidated. It was first found in *Drosophila* SL2 cells that specific protein 1 (Sp1), aryl hydrocarbon receptor (AhR) and AhR receptor nuclear translocator (Arnt) interacted with each other and bound to the GC box at the promoter to enhance the transcription of *CYP1A1* (Wang et al., 1999). In human cells, the GC box is a key *cis*-element that activates *CYP1A1* promoter activity (Zhang et al., 1998). Decreased expression of AhR in human hepatocytes inhibits the expression of *CYP1A1* (Le et al., 2010). The mechanisms underlying *CYP1A1* basal transcription remain unresolved, especially concerning the functions of Sp1 and AhR in mammalian cells.

Sp1 is expressed ubiquitously in cells and transactivates many genes by binding to the GC box at promoters through three C_2_H_2_-type zinc fingers at the C-terminus (Wierstra, 2008). The pathways via which Sp1 acts in the regulation of gene expression are versatile. One or more Sp1 molecules bind to a single site at the promoter of a gene to activate gene transcription; additionally, multiple Sp1 molecules are recruited to multiple Sp1 binding sites at the promoter to synergistically activate gene expression (Deniaud et al., 2009). Many studies confirmed that Sp1 participates in the regulation of CYP450 gene expression. As an example, Sp1 is critical for the regulation of CYP3A5 basal expression in humans and pigs (Iwano et al., 2001; Saito et al., 2001; Bombail et al., 2004). However, few studies have focused on Sp1 function in the regulation of *CYP1A1* in human cells and none have focused on porcine cells.

*CYP1A1* expression has been confirmed to be inducible by many exogenous compounds. AhR is regarded as the target transcription factor to induce *CYP1A1*. AhR is abundant in the cytoplasm, is transported to the nucleus by heterodimerization with ARNT and then binds to the xenobiotic response element (XRE) at the promoter of the gene, thereby activating gene expression (Whitelaw et al., 1993). Many exogenous compounds, such as polycyclic aromatic hydrocarbons (PAHs), biphenyls (PCBs), and halogenated aromatic hydrocarbons (HAHs), induce *CYP1A1* expression by AhR-dependent pathways (Hanlon et al., 2005; Fazili et al., 2010; Xie et al., 2018). However, several studies have found that AhR-independent pathways regulate the induction of *CYP1A1*. For example, α-naphthoflavone, an AhR-interfering agent, does not antagonize the induction of *CYP1A1* in HepG2 cells treated with TCDD (Kikuchi et al., 1998). Retinoids induced *CYP1A1* via binding to the retinoic acid response element in its promoter region (Vecchini et al., 1994). These studies indicate that the regulatory pattern of *CYP1A1* is coordinated by multiple pathways. However, the regulation of porcine *CYP1A1* remains unclear presently.

In our study, initially, we confirmed that the key regulatory elements for its basal transcription are in the proximal promoter region using truncated promoter analysis by a dual luciferase assay. Two overlapped *cis*-elements, the GC box and XRE, possibly recruiting Sp1 and AhR, respectively, transactivate basal transcription in the proximal promoter of p*CYP1A1*. Knockdown of Sp1 and AhR suggested that only Sp1 is involved in the regulation of the proximal promoter of p*CYP1A1*. Furthermore, in LLC-PK1 cells treated with an agonist and antagonist of AhR, neither activation nor inhibition of AhR activity affected the basal transcription of p*CYP1A1* in the proximal promoter. Electrophoretic mobility shift assay (EMSA) and chromatin immunoprecipitation (ChIP) confirmed that Sp1 binds to the proximal promoter of p*CYP1A1*.

## Materials and Methods

### Construction of Vectors

A 3.5-kb fragment (-3424 to +157; +1 indicates the transcriptional start site) from the 5′-flanking region of porcine *CYP1A1* (GenBank Accession No. NC_010449.5) was amplified by PCR from the genomic DNA of porcine liver tissues. This fragment was inserted into the pGL3-Basic vector at Xho I and Mlu I sites to generate the -3424-Luc plasmid. Using this plasmid as a template and the same downstream primer, a series of upstream truncated primers were used to amplify fragments of different lengths. These fragments were inserted into the pGL3-Basic vector at Xho I and Mlu I sites to generate the truncated plasmids. Using primers designed for different mutation patterns, ΩPCR was used to construct different mutation vectors using the -43-LUC plasmid as a template. The open reading frames for the expression of Arnt (GenBank Accession No. NC_010446.5), AhR (GenBank Accession No. NC_010451.4), and Sp1 (GenBank Accession No. NC_010447.4) were amplified with the corresponding primers from cDNA that was reversely transcribed from total RNA extracted from porcine liver cells. The open reading frames were inserted into the Xba I and Hind III sites of pcDNA3.1/myc-His(-) A vectors (Invitrogen). The primers used are listed in **Supplementary Table S1**.

### Cells Culture and Reagents

LLC-PK1 cells (ATCC, CL-101) were cultured in M199 medium, and COS7 cells (ATCC, CRL-1651) were cultured in DMEM medium. All media were supplemented with 10% fetal bovine serum (PAN-Biotech, Aidenbach, Germany), 1% penicillin G/streptomycin (Invitrogen), 1× SITE (Sigma), 0.1 μM dexamethasone and 0.1 μM insulin (Invitrogen). All cell lines were cultivated at 37°C, 5% CO_2_.

### RNA Isolation and Real-Time PCR

Total RNA was isolated using TRIZOL reagent (Invitrogen). Total RNA was treated with DNase I (M0303S, New England BioLabs) to remove the genomic DNA before performing reverse transcription. Thereafter, 1 μg of RNA was reversely transcribed using M-MLV Reverse Transcriptase (Promega), random primer (6mer) and 10 mM dNTPs (TaKaRa).

Real-time PCR was performed using the BioRad CFX96 real-time PCR detection system (Bio-Rad, Hercules, CA, United States) according to the manufacturer’s instructions. RT-PCR was performed in 20-μL volumes containing SYBR Green I Dye. The following parameters were used: 94.0°C for 2 min, followed by 40 cycles of 94.0°C for 20 s, 50.0°C for 30 s, and 72.0°C for 30 s. The gene expression levels were normalized using GAPDH as a control. The 2^-ΔΔC_T_^ method was used to process data and calculate the relative expression of genes. The primers used are listed in **Supplementary Table S1**.

### Western Blotting

According to the manufacturer’s instructions (Beyotime, Shanghai, China), RIPA lysis buffer containing 1 mM phenylmethylsulfonyl fluoride (PMSF) was used to extract total cellular protein. The protein concentration was adjusted using the BCA Protein Assay Kit (Pierce). Next, 30 μg of total protein was subjected to 10% polyacrylamide gel electrophoresis, followed by transfer to a PVDF membrane (Millipore). The membranes were blocked in a buffer containing 5% skim milk and 0.1% Tween 20 in Tris-buffered saline (0.15 M sodium chloride and 20 mM Tris base, pH 8.0) for 1 h at room temperature. Next, the membranes were incubated with antibodies overnight at 4°C. Secondary antibodies were incubated for 1 h at room temperature. The bands were detected using the ChemiDoc MP Imaging system (Bio-Rad, United States). The antibodies used in this work included anti-GAPDH (Santa Cruz, sc-32233), anti-Sp1 (Abcam, ab13370), anti-AhR (Cell Signaling Technology, D5S6H), and anti-CYP1A1 (Santa Cruz, sc-101828).

### Transfection and Luciferase Activity Assay

Cells were grown in 24-well plates for 24 h to 70–90% confluence. Next, 0.5 μg of the pGL3-basic plasmid containing the promoter and 2.5 ng of the pRL-CMV plasmid were transfected into cells with 1.5 μL of Lipofectamine 3000 (Invitrogen). The transfection reagent was removed, and then 500 μL of medium with 10% FBS was added to each well after 5 h. After 24 h, the cells were lysed, and the luciferase activities were measured using the Dual Luciferase Reporter Assay System (Promega) on a Turner Designs TD-20/20n luminometer (Promega). The dual luciferase reporter assay system was purchased from Promega (Madison, WI, United States). The ratio of the firefly luciferase activity to the Renilla luciferase activity represents the strength of the promoter.

### siRNA Transfection

All siRNAs were synthesized by GenePharma (Suzhou). The cells were grown in 24-well plates for 24 h to 70–90% confluence, and then 40 pmol of siRNA and plasmid were transfected into the cells with 1.5 μL of Lipofectamine 3000 (Invitrogen). Non-targeting siRNA (scramble) was used as the negative control. The transfection reagent was removed, and 500 μL of medium with 10% FBS was added to each well after 5 h. After 24 h, the cells were lysed, and the luciferase activities were measured. The siRNA sequences used are listed in **Supplementary Table S1**.

### Electrophoresis Mobility Shift Assay (EMSA)

For overexpression experiments, COS-7 cells were transfected with the constructs of the Sp1 or AhR gene. The Nuclear Extraction Kit (Beyotime, Haimen, China) was used to extract overexpressed nuclear protein from COS7 cells. EMSA was performed using the EMSA Assay Kit (Beyotime, Haimen, China). The mixtures containing unlabeled probe, nuclear extract (4 μg) and binding buffer were preincubated at 23°C for 10 min. Next, 0.1 pmol of the biotinylated probe was added and incubated at 23°C for 20 min in a total volume of 9 μL. The mixture was subjected to electrophoresis in 4.5% non-denaturing polyacrylamide [acrylamide/bisacrylamide 29:1 (v/v)] gels, followed by the addition of bromophenol blue in a blank hole as an indicator. The gel was transferred to a nylon membrane (+) after the bromophenol blue appeared at approximately 2 cm from the bottom of the gel. The nylon membrane (+) was crosslinked for 2 min under UV light (254 nm). The nylon membrane (+) was then processed with the EMSA Assay Kit (Beyotime, Haimen, China), followed by the detection by chemiluminescence (Millipore, Bedford, MA, United States). For competition experiments, 100-fold molar excess of unlabeled probe was used. For supershift experiments, 1 μL of anti-Sp1 (Abcam, ab13370), and anti-AhR (Cell Signaling Technology, D5S6H) were used.

### Chromatin Immune-Precipitation (ChIP)

According to the manufacturer’s instructions (Cell Signaling Technology, #9003, United States), the enrichment of Sp1 and AhR in the *CYP1A1* promoter in LLC-PK1 cells was analyzed. The antibodies used in this work included anti-histone H3 (Cell Signaling Technology, D2B12), anti-Sp1 (Abcam, ab13370), and anti-AhR (Cell Signaling Technology, D5S6H). The primers used are listed in **Supplementary Table S1**.

### Statistical Analysis

All experiments were performed independently at least three times. All the data are represented as the means ± standard deviation (SD). Statistical significance was assessed by ANOVA. Values of *P* < 0.05 were considered statistically significant.

## Results

### Identification of *cis*-Elements in the Porcine *CYP1A1* Promoter That Potentially Regulate *CYP1A1* Expression

**FIGURE 1:**
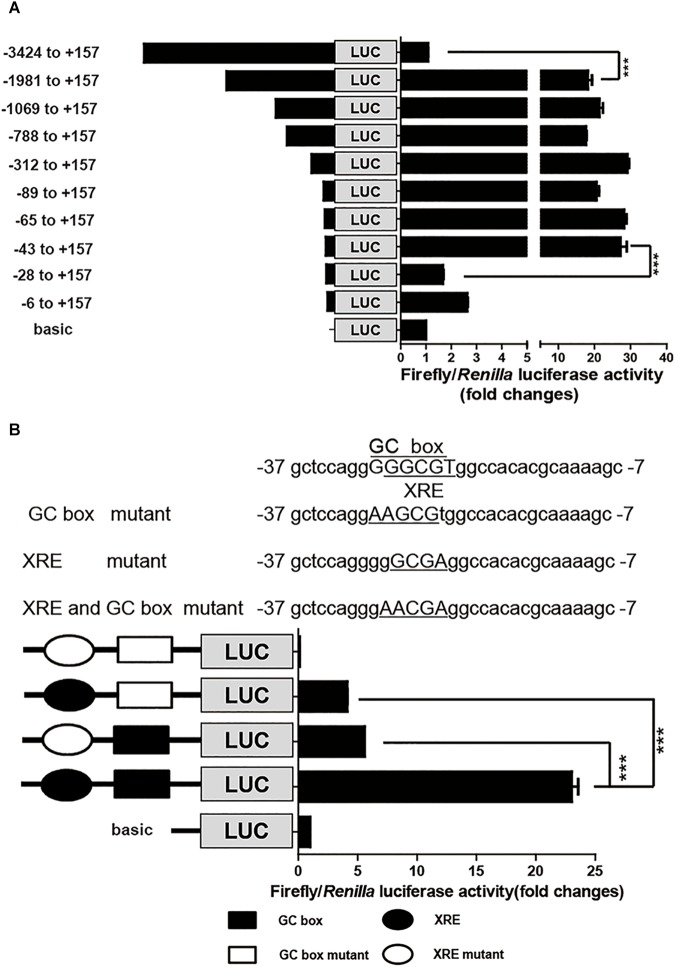
The proximal GC box and XRE determine p*CYP1A1* basal expression. **(A)** Deletion analysis of the p*CYP1A1* promoter in LLC-PK1 cells. pGL3-basic containing different lengths of the p*CYP1A1* promoter fragments and pRL-CMV plasmid were transfected into LLC-PK1 cells, and the luciferase activities were measured, as described in section “Materials and Methods.” Basic represents empty vector pGL3-basic, which served as the control. The values on the right show the ratio of the luciferase activity of the promoter fragment to the luciferase activity of pGL3-basic. The left diagrams show the deletion pattern of the p*CYP1A1* promoter. The number indicates the position of the fragment (base pair) at the transcription start site. **(B)** Mutation analysis of the proximal p*CYP1A1* promoter in LLC-PK1 cells. The mutation pattern is shown above and represent the mutations that are introduced into the –37 to –7 promoter region to destroy the XRE and GC box. The mutated bases are underlined. The left diagram shows the mutation pattern of the p*CYP1A1* promoter. The value on the right shows the ratio of the luciferase activity of the promoter fragment to the luciferase activity of pGL3-basic. The experiment was repeated three times. Error bars represent the standard deviation of three replicates. ^∗∗∗^*P* < 0.001.

To identify the *cis*-elements that regulate *CYP1A1* expression in the porcine *CYP1A1* promoter, DNA fragments with different lengths, spanning from the ATG of the p*CYP1A1* gene to -3424 bp upstream were fused upstream of the firefly luciferase gene. Each promoter plasmid and reference plasmid, pRL-CMV harboring the Renilla luciferase gene, were co-transfected into LLC-PK1 cells. Normalized luciferase activity indicates the strength of the promoter for transcription activities. As shown in **Figure 1A**, the removal of DNA fragments from -3424 to -1981 bp led to the apparent incremental change in firefly luciferase activity, suggesting there are negative regulatory elements in this region to inhibit the basal transcription of porcine p*CYP1A1*. More importantly, the firefly luciferase activity was apparently decreased when DNA fragments from -43 to -28 bp were deleted, from 27- to 1.7-fold that of the normalized basic control. This suggested that the proximal promoter is the key region for p*CYP1A1* transactivation and contains positive regulatory elements between -43 and -28 bp (**Figure 1A**).

The MatInspector program^1^ was used to analyze the candidate *cis*-elements in the proximal promoter DNA sequence regarding transcriptional regulation. Two *cis*-elements, GC box and XRE, were found in this -43 to -28 bp region, and they probably play important roles in p*CYP1A1* expression. These two *cis*-elements overlapped in the region, and the core DNA sequences only have one base difference. Therefore, direct deletion of one *cis-*element to confirm its role in the p*CYP1A1* promoter transactivation is not applicable. To verify the critical roles for the two *cis*-elements in basal transcription, the key binding sites were mutated, and then the strengths of the mutated promoters were analyzed by the dual luciferase assay. The relative luciferase activity was decreased by 4.5-fold in the plasmid with the mutated GC box and 5.6-fold in the plasmid with mutated XRE (**Figure 1B**). This implied either that both *cis*-elements were essential for the basal transcription or the mutation abolished the function of non-mutated *cis* element. The double mutations of the GC box and XRE in the promoter region caused the complete loss of basal transactivity because the relative luciferase activity was even lower than that of the reference control. Our data suggest that the mutation of key binding sites may lead to a combination of repressor in this region. Therefore, a mutation assay cannot confirm whether either one of these two *cis* elements or both are the key players.

### Sp1 Transactivates the Proximal Promoter Activity of p*CYP1A1*

**FIGURE 2:**
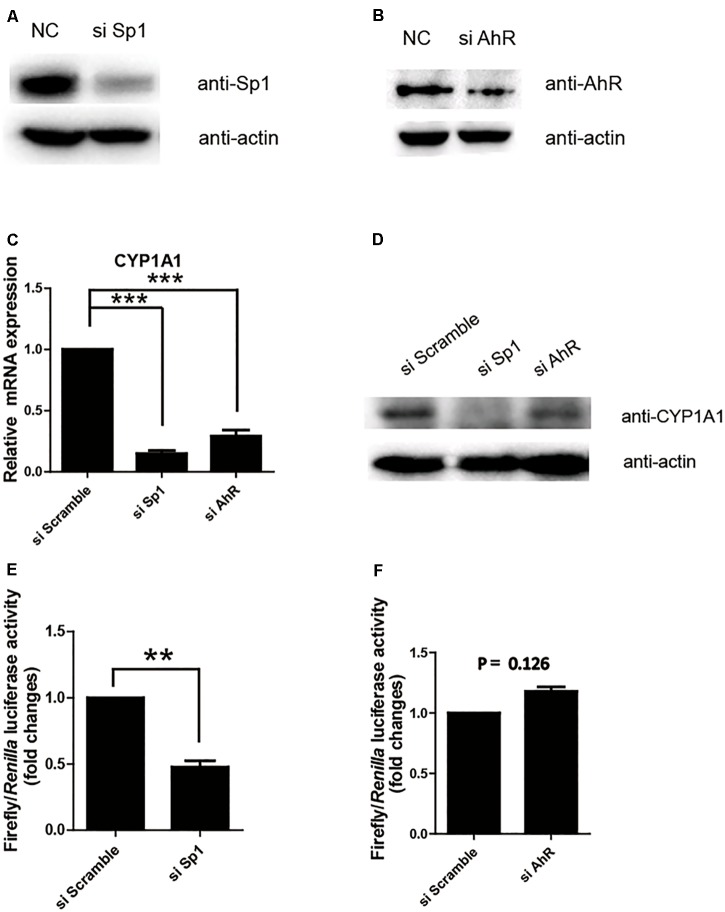
Sp1 is required for the basal transactivation of the proximal promoter of p*CYP1A1*. **(A,B)** Western blotting confirmed Sp1 or AhR protein levels after siRNA transfection in LLC-PK1 cells. **(C,D)** mRNA and protein levels of pCYP1A1 in the cells with knocked down Sp1 or AhR. RT-PCR confirmed the p*CYP1A1* mRNA levels after transfection of siRNA. Western blotting confirmed the pCYP1A1 protein levels after transfection of siRNA. **(E,F)** Sp1 but not AhR affects the proximal promoter activity of p*CYP1A1*. The siRNA, pRL-CMV plasmid and -43-Luc plasmid were co-transfected into LLC-PK1 cells. Non-targeting siRNA (NC) was used as the negative control. The promoter activity of the -43-Luc plasmid was measured. The value on the ordinate indicates the ratio of the value of the promoter activity transferred to the si Sp1 or si AhR to the value of the initiation activity transferred to the NC. The experiment was repeated three times. Error bars represent the standard deviation of three replicates. ^∗∗^*P* < 0.01 and ^∗∗∗^*P* < 0.001.

Next, we further confirmed whether Sp1 or AhR plays a key transactivation role in the proximal promoter region by measuring the relative luciferase activity of the (-43/+157) promoter in the cell lines with the knocking down of Sp1 or AhR. As shown in **Figures 2A,B**, siRNA successfully repressed the expression of Sp1 or AhR, respectively. The protein and mRNA levels of pCYP1A1 following the knocking down of Sp1 or AhR were apparently decreased (**Figures 2C,D**). This suggested that both transcription factors play important regulatory roles in p*CYP1A1* expression. However, when only measuring the transcription in the proximal promoter using the relative luciferase assay, a twofold decrease in transactivity was observed in the Sp1-knockdown cells but not in AhR-knockdown cells (**Figures 2E,F**). These data demonstrated that Sp1, rather than AhR, affects the regulation of the p*CYP1A1* proximal promoter. Taken together, the data proved that, in the proximal region of the p*CYP1A1* promoter, Sp1 transactivates the basal expression of p*CYP1A1*, and AhR does not contribute to the proximal DNA transactivation of p*CYP1A1*.

### AhR Does Not Affect the Proximal Promoter Activity of p*CYP1A1*

**FIGURE 3:**
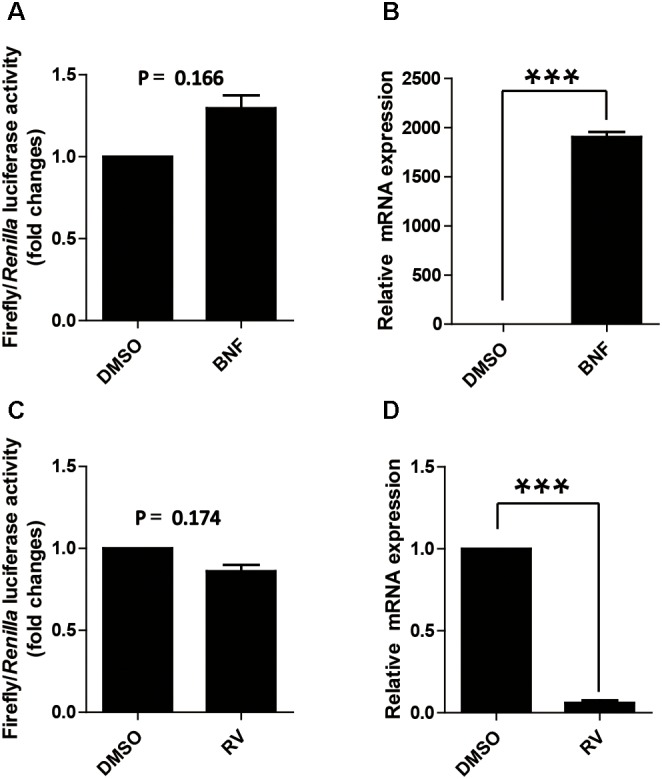
Effect of the agonist and antagonist for AhR to treat cells on promoter activity of p*CYP1A1.* **(A)** Effect of β-naphthoflavone on -43-Luc plasmid promoter activity. “BNF” indicates β-naphthoflavone treatment at a concentration of 2 μM for 24 h. DMSO treated cells were used as the control. The values on the ordinate represent the ratio of the activity of the promoter after BNF treatment to the value of the activity of the DMSO treated promoter. **(B)** The relative quantified mRNA level of *CYP1A1* in LLC-PK1 Cells treated with BNF for 24 h. DMSO-treated cells were used as the control. In the control cells, p*CYP1A1* relative to glyceraldehydes-3-phosphate dehydrogenase (GADPH) mRNA level was set to 1. **(C)** Effect of resveratrol on -43-Luc plasmid promoter activity. “RV” means resveratrol used at a concentration of 180 μM for 24 h. DMSO-treated cells were used as the control. The values on the ordinate represent the ratio of the activity of the promoter after RV treatment to the value of the activity of the DMSO-treated promoter. **(D)** The relative quantified mRNA level of *CYP1A1* in LLC-PK1 cells treated with 180 μM RV for 24 h. DMSO-treated cells served as the control. In the control cells, p*CYP1A1* relative to the mRNA level of GAPDH was set to 1. The experiment was repeated three times. Error bars represent the standard deviation of three replicates. ^∗∗∗^*P* < 0.001.

As mentioned above, AhR expression in cells does not affect the proximal promoter activity of p*CYP1A1*, which is contradicted to the previous reported that AhR affects the activity of the *CYP1A1* proximal promoter (Kobayashi et al., 1996). Two drugs, β-naphthoflavone and resveratrol, were used to further address the function of AhR in the proximal promoter of p*CYP1A1*. β-Naphthoflavone, as an agonist for AhR, has been reported to induce p*CYP1A1* expression via the translocation of cytoplasmic AhR to the nucleus (Hankinson, 1995; Kikuchi et al., 1998). In β-naphthoflavone-treated LLC-PK1 cells, p*CYP1A1* mRNA was increased by 2000-fold relative to the level in untreated cells (**Figure 3B**). Nevertheless, the (-43/+157) promoter was not changed significantly compared with the relative luciferase activity in β-naphthoflavone-treated LLC-PK1 cells with the activity in untreated control at 24 h (**Figure 3A**). Resveratrol inhibits the binding of AhR to the promoter DNA, thereby AhR activation of gene expression is inhibited. When (-43/+157) promoter plasmids were transfected into cells, similarly, there was no significant change in the relative luciferase activity in resveratrol-treated cells for 24 h (**Figure 3C**). In resveratrol-treated LLC-PK1 cells, p*CYP1A1* mRNA was decreased by 18-fold relative to that in untreated cells (**Figure 3D**).

The agonist and antagonist for AhR do not significantly affect the relative luciferase activity of the (-43/+157) promoter plasmid. These data further confirmed that the transcription factor that affects the (-43/+ 157) promoter activity is not AhR.

### Sp1 Binding to the GC Box Affects the Activity of the p*CYP1A1* Promoter

**FIGURE 4:**
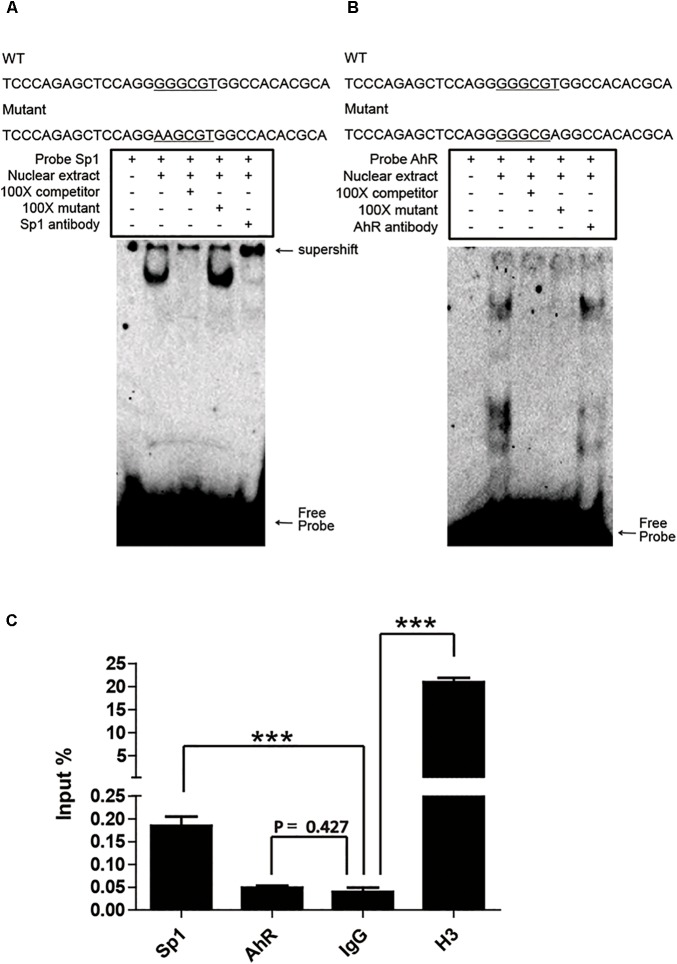
Sp1 binds to the GC box and then affects the activity of the p*CYP1A1* promoter. **(A)** EMSA was used to confirm that Sp1 binds to the GC box at the proximal p*CYP1A1* promoter. **(B)** EMSA was used to confirm that AhR does not bind to XRE at the proximal p*CYP1A1* promoter. The mutated bases are underlined. For the competition experiments, 100-fold molar excess of the unlabeled probe was used. The DNA protein complexes and supershifts are indicated by arrows. **(C)** ChIP was used to confirm that Sp1 or AhR binds to *cis* elements at the proximal p*CYP1A1* promoter. IgG was used as the negative control. Histone H3 antibody served as the positive control. The experiment was repeated three times. Error bars represent the standard deviation of three replicates.^∗∗∗^*P* < 0.001.

Sp1 and AhR bind to the GC box and XRE, respectively, which are located in the proximal promoter region of p*CYP1A1*. As mentioned above, it is not applicable to verify the key role of these two *cis*-elements in the regulation of p*CYP1A1* by deletion or mutation of the *cis*-elements. Therefore, we initially confirmed the binding of Sp1 or AhR to the DNA motif *in vitro* by EMSA. Sp1 or AhR was overexpressed in COS-7 cells. For Sp1, when the probe was incubated with the nuclear protein, probe migration would lag to form a band. A supershift occurred after the addition of the Sp1 antibody, and the band disappeared after the addition of a 100-fold molar excess of the unlabeled probe, but not after the addition of a 100-fold molar excess of unlabeled and mutated probe (**Figure 4A**). For AhR, it is dubious that AhR bound with the probe, and no supershift band was observed (**Figure 4B**). These data indicated that Sp1 binds at the position of -37 to -7 bp of the p*CYP1A1* promoter and then transactivates the expression of p*CYP1A1*. ChIP was used to verify whether Sp1 and AhR bind to the p*CYP1A1* proximal promoter. The binding of Sp1 to the porcine *CYP1A1* proximal promoter was fourfold greater than that of the negative control, while the binding of AhR to the porcine *CYP1A1* proximal promoter was not significantly different from that of the negative control (**Figure 4C**). Taken together, those data proved that Sp1, but not AhR, binds to the proximal region of the p*CYP1A1* promoter.

## Discussion

Cytochrome P450 is a superfamily of enzymes known to metabolize exogenous compounds. Among them, CYP1A1 is well-known for its ability to metabolize drugs and other xenobiotic substances (Guengerich et al., 1991). The pig is an animal model that can substitute for humans in pharmacological and toxicological studies. The amino acid sequence similarity of CYP1A1 between pigs and humans exceeds 80%, indicating that they have the same metabolic characteristics. Presently, the regulatory mechanism of *CYP1A1* in pigs is not clear. To analyze the conservation of the regulatory mechanisms of *CYP1A1* in humans and pigs, it is necessary to clarify the expression regulation mechanism of p*CYP1A1.*

Truncation analysis of the p*CYP1A1* promoter DNA showed that the proximal region (-43 to -28) is the key determinant region for p*CYP1A1* expression. In this region, two *cis*-elements were identified, but the core binding sequence is overlapped. We also found that these two *cis*-elements at the human *CYP1A1* proximal promoter overlap and play important roles in human *CYP1A1* basal transcription (Internal communication). Furthermore, by knocking down either Sp1 or AhR via siRNA, the expression levels of pCYP1A1 at the mRNA and protein levels were significantly decreased, suggesting that both are important in p*CYP1A1* basal transcription. However, using EMSA and ChIP, it was confirmed that only Sp1 binds to the GC box in the proximal promoter. Instead, AhR did not bind to this region, although the expression of p*CYP1A1* was drastically decreased in si-AhR cells. This finding suggested that, in the proximal promoter region, Sp1 determines the strength of transcription. AhR probably affected the expression of p*CYP1A1* in this proximal region, which harbors other functional XRE sites.

Studies have shown that AhR is an important transcription factor that participates in the expression of *CYP1A1*. The high induction of *CYP1A1* expression by many exogenous compounds like PAHs, PCBs, and HAHs relies on the AhR-dependent pathway (Ma et al., 2000; Monk et al., 2003; Uno et al., 2004). Among them, the mechanism by which TCDD induces the expression of *CYP1A1* through AhR is clear. TCDD increases the level of AhR in the nucleus and induces the expression of *CYP1A1* in humans and pigs (Xu et al., 2000; Hombachklonisch et al., 2006). In addition, AhR is a methylation-sensitive transcription factor. TCDD induces a decrease in DNA methylation in the *Cyp1a1* promoter region of mice, thereby enhancing the expression of *Cyp1a1* via an AhR-dependent pathway (Amenya et al., 2016).

In humans, AhR is important for the basal regulation of CYP1A (Le et al., 2010), but how AhR activates the p*CYP1A1* basal promoter activity is unclear. In our study, AhR was also important for the basal regulation of CYP1A1 in pigs. Intriguingly, we found that the DNA region that regulates p*CYP1A1* expression is mainly located proximal to the transcription start site, whereas XRE in this region does not participate in the regulation of p*CYP1A1*. These data are inconsistent with previous studies. Therefore, we performed promoter strength analysis under the condition that AhR was regulated by its agonist or antagonist to further address the effect of AhR on the proximal promoter of p*CYP1A1*. β-Naphthoflavone has been reported to enhance gene expression by activating AhR, and resveratrol reduces gene expression by inhibiting AhR (Lee and Safe, 2001; Fujita and Mtetsuhashi, 2010). Indeed, β-naphthoflavone and resveratrol treatment of LLC-PK1 cells increased or decreased the expression of p*CYP1A1* mRNA, suggesting that AhR binds to the promoter of p*CYP1A1.* Using the MatInspector program to analyze the 3.5-kb promoter DNA, 12 XREs were predicted to be in the promoter region of p*CYP1A1*, and 10 of them were located in the region of -819 to -2494 nt upstream of the transcription start site. However, when proximal promoter analysis was applied, it showed that neither activation nor deactivation of AhR showed significant effects on the activity of the p*CYP1A1* proximal promoter. Our results demonstrate that AhR regulates the expression of p*CYP1A1*, but it is not involved in the regulation of the proximal promoter of p*CYP1A1*.

Considering only the regulation of this proximal promoter, the luciferase assay clearly suggested that only Sp1 transactivates the basal expression of p*CYP1A1* rather than AhR. Sp1 participates in the expression of many CYP450 genes, such as *CYP1A1*, *CYP1B1*, *CYP2J2*, *CYP3A29*, and *CYP3A46* (Zhang et al., 1998; Tsuchiya et al., 2003; Spiecker et al., 2004; Dong et al., 2015; Liu et al., 2016). The amino acid sequence for Sp1 in pigs and humans has more than 90% identity similarity, leading us to believe that Sp1 might have a conserved regulatory function in *CYP1A1* expression. In humans, Sp1 activates the *CYP1A1* promoter (Zhang et al., 1998), and phosphorylation of Sp1 regulates *CYP1A1* expression (Shimoyama et al., 2014). We also found that the GC box at the proximal promoter of p*CYP1A1* is the main *cis*-element that plays a regulatory role by recruiting Sp1.

CYP1A1 not only functions to metabolize exogenous compounds but also functions in the metabolism of endogenous chemicals, such as polyunsaturated fatty acids (Westphal et al., 2011; Chi et al., 2016). Comparing the pattern of CYP1A1 expression regulation in pigs and humans, we can see that Sp1 is very conservative in the regulation of CYP1A1. This shows the importance of Sp1 for pigs and humans in the gene regulation such as CYP1A1.

In summary, by analyzing the porcine *CYP1A1* promoter activity, we found that the proximal promoter is very important for the regulation of p*CYP1A1* expression. Two *cis* elements were found in the proximal promoter region: XRE and GC box with the binding of Sp1 and AhR, respectively. However, only Sp1 plays a transactivation role in the proximal promoter region of p*CYP1A1*. AhR also regulates the expression of p*CYP1A1*, but it does not function at the proximal promoter.

## Author Contributions

XX, JJ, WY, and RC conducted the experiments and data analysis. XX and JW wrote this article. YD and JW conceived the study.

## Conflict of Interest Statement

The authors declare that the research was conducted in the absence of any commercial or financial relationships that could be construed as a potential conflict of interest.

## Footnotes

1 http://www.genomatix.de/online_help/help_matinspector/matinspector_help.html
