# Supplementary material for: Sp1, Instead of AhR, Regulates the Basal Transcription of Porcine CYP1A1 at the Proximal Promoter
Source: Front Pharmacol. 2018 Aug 17;9:927. doi: 10.3389/fphar.2018.00927 (PMC6107784; doi:10.3389/fphar.2018.00927)
Supplement: Supplementary file 1 [file Table_1.docx]

Supplementary Table S1. Primers used in this study.

| Primer name | | sequence（5’-3’） | |
| --- | --- | --- | --- |
| Truncated analysis | | | |
| （-3424/+157）-LUC-F | | CGACGCGTTCTGGGCTCTGCTTTCCCTGGTCAT | |
| （-1981/+157）-LUC-F | | CGACGCGTCTCAGCAAGGTGCTCCCCTGAACTG | |
| （-1069/+157）-LUC-F | | CGACGCGTTGGCCCTTTAAGAGCCCCGCCTCC | |
| （-1040/+157）-LUC-F | | CGACGCGTCCCTCCCCACCCCCCTCGCGTGACT | |
| （-1033/+157）-LUC-F | | CGACGCGTCACCCCCCTCGCGTGACTGTATCGG | |
| （-980/+157）-LUC-F | | CGACGCGTTGGGTGGCTGCGCGGGCCTCCGGGC | |
| （-908/+157）-LUC-F | CGACGCGTGGGATGGGCCGCGTGACCTCTGCCC | |  |
| （-901/+157）-LUC-F | | CGACGCGTGCCGCGTGACCTCTGCCCCCTGGAG | |
| （-844/+157）-LUC-F | | CGACGCGTGAGGCATCGGCACGGGACTCCCTCC | |
| （-788/+157）-LUC-F | CGACGCGTGGCCCTCCCTCGCCGCCCAATTTCT | |  |
| （-312/+157）-LUC-F | | CGACGCGTGCCACCCCATCACCCAATAGCCTCT | |
| （-89/+157）-LUC-F | | CGACGCGTGAAGACCGTCCCCCCCAACCCCAGG | |
| （-65/+157）-LUC-F | | CGACGCGTGCTGCCCCTCCCTCTGTCCCATCCC | |
| （-43/+157）-LUC-F | | CGACGCGTCCCAGAGCTCCAGGGGGCGTGGC | |
| （-28/+157）-LUC-F | | CGACGCGTGGCGTGGCCACACGCAAAAGCTGCC | |
| （-6/+157）-LUC-F | | CGACGCGTGCCTATAAAGGTGGCAGTGCC | |
| LUC-R | | CCGCTCGAGGATCGGCGTAGAGATTCCAAGGTGGC  TGCTGCGGCTCTGAGGGCTGCAGAGCTGAGCT | |
| Mutation analysis | | | |
| AhR mutant F | | AGCTCCAGGGGGCGAGGCCACACGCAAAAG | |
| AhR mutant R | | CTTTTGCGTGTGGCCTCGCCCCCTGGAGCT | |
| GC box mutant F | | TGCGTGTGGCCACGCTTCCTGGAGCTCTGGGA | |
| GC box mutant R | | TCCCAGAGCTCCAGGAAGCGTGGCCACACGCA | |
| AhR and GC box mutant F | | TACGCGTCCCAGAGCTCCAGGAAGCAAGGCCAC  ACGCAAAAGCTGCCTA | |
| AhR and GC box mutant R | | TAGGCAGCTTTTGCGTGTGGCCTTGCTTCCTGG  AGCTCTGGGACGCGTA | |
| RT-PCR | | | |
| CYP1A1-F | | ATCCTGGAGCTCTTCCGAC | |
| CYP1A1-R | | GGTATGATCCCTCAGGCTTG | |
| *GAPDH*-F | | GTCGGTTGTGGACCTGAC | |
| *GAPDH*-R | | TGGTCGTTGAGGGCAATG | |
| ChIP | | | |
| F | | GAAGACCGTCCCCCCCAACCCCAGG | |
| R | | TGAAGGCACTGCCACCTTTA | |
| si RNA sequence | | | |
| si AhR | | CCUAUGUGGAAGAUUCUUUTT | |
| si Sp1 | | GCAGCUACCUUGACUCCUATT | |
| Overexpression of transcription factors | | | |
| AhR-F | | ATGAACAGCAGCAGCGCCAACATCACCTACGCCAG | |
| AhR-R | | TTACAGGAATCCACTAGATGTCAAGTCAGGGAAAG  TTCTGGCTTCTGACG | |
| Arnt-F | | ATGGCGGCGACTACTGCTAACCCCGAAATG | |
| Arnt-R | | CTATTCTGAAAAAGAGGGAAACATAGTTAG  ATCAGGAAATTCTTCATTGT | |
| Sp1-F | | ATGAGCGACCAAGATCACTCCATG | |
| Sp1-R | | TCAGAAGCCATTGCCACTGATATTA | |
